# Predicting COVID-19 Vaccination Uptake Using a Small and Interpretable Set of Judgment and Demographic Variables: Cross-Sectional Cognitive Science Study

**DOI:** 10.2196/47979

**Published:** 2024-03-18

**Authors:** Nicole L Vike, Sumra Bari, Leandros Stefanopoulos, Shamal Lalvani, Byoung Woo Kim, Nicos Maglaveras, Martin Block, Hans C Breiter, Aggelos K Katsaggelos

**Affiliations:** 1 Department of Computer Science University of Cincinnati Cincinnati, OH United States; 2 Department of Electrical and Computer Engineering Northwestern University Evanston, IL United States; 3 School of Medicine Faculty of Health Sciences Aristotle University of Thessaloniki Thessaloniki Greece; 4 Integrated Marketing Communications Medill School Northwestern University Evanston, IL United States; 5 Department of Psychiatry Massachusetts General Hospital Harvard School of Medicine Boston, MA United States; 6 Department of Computer Science Northwestern University Evanston, IL United States; 7 Department of Radiology Northwestern University Evanston, IL United States

**Keywords:** reward, aversion, judgment, relative preference theory, cognitive science, behavioral economics, machine learning, balanced random forest, mediation, moderation, mobile phone, smartphone

## Abstract

**Background:**

Despite COVID-19 vaccine mandates, many chose to forgo vaccination, raising questions about the psychology underlying how judgment affects these choices. Research shows that reward and aversion judgments are important for vaccination choice; however, no studies have integrated such cognitive science with machine learning to predict COVID-19 *vaccine uptake*.

**Objective:**

This study aims to determine the predictive power of a small but interpretable set of judgment variables using 3 machine learning algorithms to predict COVID-19 *vaccine uptake* and interpret what profile of judgment variables was important for prediction.

**Methods:**

We surveyed 3476 adults across the United States in December 2021. Participants answered demographic, COVID-19 *vaccine uptake* (ie, whether participants were fully vaccinated), and COVID-19 precaution questions. Participants also completed a picture-rating task using images from the International Affective Picture System. Images were rated on a Likert-type scale to calibrate the degree of liking and disliking. Ratings were computationally modeled using relative preference theory to produce a set of graphs for each participant (minimum *R*^2^>0.8). In total, 15 judgment features were extracted from these graphs, 2 being analogous to risk and loss aversion from behavioral economics. These judgment variables, along with demographics, were compared between those who were fully vaccinated and those who were not. In total, 3 machine learning approaches (random forest, balanced random forest [BRF], and logistic regression) were used to test how well judgment, demographic, and COVID-19 precaution variables predicted *vaccine uptake*. Mediation and moderation were implemented to assess statistical mechanisms underlying successful prediction.

**Results:**

Age, income, marital status, employment status, ethnicity, educational level, and sex differed by *vaccine uptake* (Wilcoxon rank sum and chi-square *P*<.001). Most judgment variables also differed by *vaccine uptake* (Wilcoxon rank sum *P*<.05). A similar area under the receiver operating characteristic curve (AUROC) was achieved by the 3 machine learning frameworks, although random forest and logistic regression produced specificities between 30% and 38% (vs 74.2% for BRF), indicating a lower performance in predicting unvaccinated participants. BRF achieved high precision (87.8%) and AUROC (79%) with moderate to high accuracy (70.8%) and balanced recall (69.6%) and specificity (74.2%). It should be noted that, for BRF, the negative predictive value was <50% despite good specificity. For BRF and random forest, 63% to 75% of the feature importance came from the 15 judgment variables. Furthermore, age, income, and educational level mediated relationships between judgment variables and *vaccine uptake*.

**Conclusions:**

The findings demonstrate the underlying importance of judgment variables for vaccine choice and uptake, suggesting that vaccine education and messaging might target varying judgment profiles to improve uptake. These methods could also be used to aid vaccine rollouts and health care preparedness by providing location-specific details (eg, identifying areas that may experience low vaccination and high hospitalization).

## Introduction

### Background

In early 2020, the COVID-19 pandemic wreaked havoc worldwide, triggering rapid vaccine development efforts. Despite federal, state, and workplace vaccination mandates, many individuals made judgments against COVID-19 vaccination, leading researchers to study the psychology underlying individual vaccination preferences and what might differentiate the framework for judgment between individuals who were fully vaccinated against COVID-19 and those who were not (henceforth referred to as *vaccine uptake*). A better understanding of these differences in judgment may highlight targets for public messaging and education to increase the incidence of choosing vaccination.

### Prior Work

Multiple studies have sought to predict an individual’s intention to receive a COVID-19 vaccine or specific variables underlying vaccination choices or mitigation strategies [1-7], but few have predicted *vaccine uptake*. One such study used 83 sociodemographic variables (with education, ethnicity, internet access, income, longitude, and latitude being the most important predictors) to predict *vaccine uptake* with 62% accuracy [8], confirming both the importance and limitations of these variables in prediction models. Other studies have compared demographic groups between vaccinated and nonvaccinated persons; Bulusu et al [9] found that young adults (aged 18-35 years), women, and those with higher levels of education had higher odds of being vaccinated. In a study of >12 million persons, the largest percentage of those who initiated COVID-19 vaccination were White, non-Hispanic women between the ages of 50 and 64 years [10]. Demographic variables are known to affect how individuals judge what is rewarding or aversive [11,12] yet are not themselves variables quantifying how individuals make judgments that then frame decisions.

**Figure 1:**
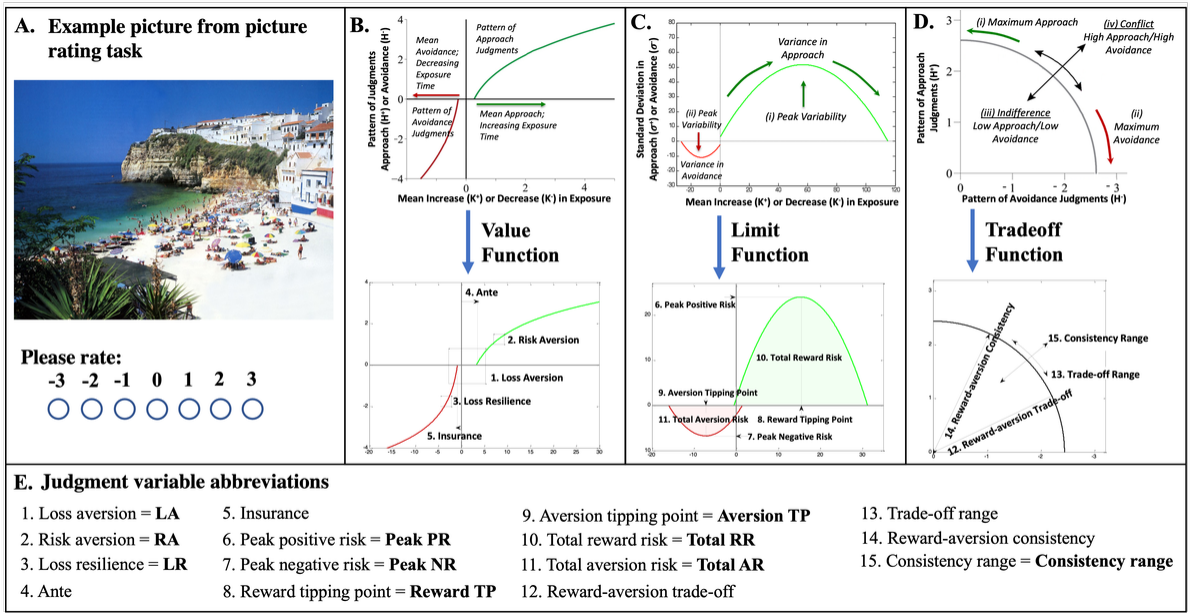
Picture-rating task and judgment variable extraction. (A) An example picture from the picture-rating task in which participants were asked to rate how much they liked or disliked an image on a scale from −3 (dislike very much) to +3 (like very much), with 0 being neutral. (B) Visual representation of the x-y plane for relative preference theory (RPT) value function fitting and resulting features extracted. (C) Visual representation of the x-y plane for RPT limit function fitting and resulting features extracted. (D) Visual representation of the x-y plane for RPT trade-off function fitting and resulting features extracted. (E) Each of the 15 features and their abbreviated terms.

Judgment reflects an individual’s preferences, or the variable extent to which they approach or avoid events in the world based on the rewarding or aversive effects of these events [13-15]. The definition of preference in psychology differs from that in economics. In psychology, preferences are associated with “wanting” and “liking” and are framed by judgments that precede decisions, which can be quantified through reinforcement reward or incentive reward tasks [12,16-21]. In economics, preferences are relations derived from consumer choice data (refer to the axioms of revealed preference [22]) and reflect choices or decisions based on judgments that place value on behavioral options. Economist Paul Samuelson noted that decisions are “assumed to be correlative to desire or want” [23]. In this study, we focused on a set of variables that frame judgment, with the presumption that judgments precede choices [12,20]. Variables that frame judgment can be derived from tasks using operant key-pressing tasks that quantify “wanting” [24-33] or simple rating tasks that are analogous to “liking” [20,34]. Both operant keypress and rating tasks measure variables that quantify the average (mean) magnitude (*K*), variance (*σ*), and pattern (ie, Shannon entropy [*H*]) of reward and aversion judgments [35]. We refer to this methodology and the multiple relationships between these variables and features based on their graphical relationships as relative preference theory (RPT; Figure 1) [18,36]. RPT has been shown to produce discrete, recurrent, robust, and scalable relationships between judgment variables [37] that produce mechanistic models for prediction [33], and which have demonstrated relationships to brain circuitry [24-27,30] and psychiatric illness [28]. Of the graphs produced for RPT, 2 appear to resemble graphs derived with different variables in economics, namely, prospect theory [38] and the mean-variance function for portfolio theory described by Markowitz [39]. Given this graphical resemblance, it is important to note that RPT functions quantifying value are not the same as standard representations of preference in economics. Behavioral economic variables such as loss aversion and risk aversion [38,40-51] are not to be interpreted in the same context given that both reflect biases and bounds to human rationality. In psychology, they are grounded in judgments that precede decisions, whereas in economics, they are grounded in consumer decisions themselves. Going forward, we will focus on judgment-based loss aversion, representing the overweighting of negative judgments relative to positive ones, and judgment-based risk aversion, representing the preference for small but certain assessments over larger but less certain ones (ie, assessments that have more variance associated with them) [38,40-51]. Herein, loss aversion and risk aversion refer to ratings or judgments that precede decisions.

A number of studies have described how risk aversion and other judgment variables are important for individual vaccine choices and hesitancies [52-58]. Hudson and Montelpare [54] found that risk aversion may promote vaccine adherence when people perceive contracting a disease as more dangerous or likely. Trueblood et al [52] noticed that those who were more risk seeking (as measured via a gamble ladder task) were more likely to receive the vaccine even if the vaccine was described as expedited. Wagner et al [53] described how risk misperceptions (when the actual risk does not align with the perceived risk) may result from a combination of cognitive biases, including loss aversion. A complex theoretical model using historical vaccine attitudes grounded in decision-making has also been proposed to predict COVID-19 vaccination, but this model has not yet been tested [59]. To our knowledge, no study has assessed how well a model comprising variables that reflect reward and aversion judgments predicts *vaccine uptake*.

### Goal of This Study

Given the many vaccine-related issues that occurred during the COVID-19 pandemic (eg, vaccine shortages, hospital overload, and vaccination resistance or hesitancy), it is critical to develop methods that might improve planning around such shortcomings. Because judgment variables are fundamental to vaccine choice, they provide a viable target for predicting *vaccine uptake*. In addition, the rating methodology used to quantify variables of judgment is independent of methods quantifying *vaccine uptake* or intent to vaccinate, limiting response biases within the study data.

In this study, we aimed to predict COVID-19 *vaccine uptake* using judgment, demographic, and COVID-19 precaution (ie, behaviors minimizing potential exposure to COVID-19) variables using multiple machine learning algorithms, including logistic regression, random forest, and balanced random forest (BRF). BRF was hypothesized to perform best given its potential benefits with handling class imbalances [60], balancing both recall and specificity, and producing Gini scores that provide relative variable importance to prediction. In this study, the need for data imbalance techniques was motivated by the importance of the specificity metric, which would reflect the proportion of participants who did not receive full vaccination; without balancing, the model might not achieve similar recall and specificity values. When there is a large difference between recall and specificity, specificity might instead reflect the size of the minority class (those who did not receive full vaccination). In general, random forest approaches have been reported to have benefits over other approaches such as principal component analysis and neural networks, in which the N-dimensional feature space or layers (in the case of neural networks) are complex nonlinear functions, making it difficult to interpret variable importance and relationships to the outcome variable. To provide greater certainty about these assumptions, we performed logistic regression in parallel with random forest and BRF. The 3 machine learning approaches used a small feature set (<20) with interpretable relationships to the predicted variable. Such interpretations may not be achievable in big data approaches that use hundreds to thousands of variables that seemingly add little significance to the prediction models. Interpretation was facilitated by (1) the Gini importance criterion associated with BRF and random forest, which provided a profile of the judgment variables most important for prediction; and (2) mediation and moderation analyses that offered insights into statistical mechanisms among judgment variables, demographic (contextual) variables, and *vaccine uptake*. Determining whether judgment variables are predictive of COVID-19 *vaccine uptake* and defining which demographic variables facilitate this prediction presents a number of behavioral targets for vaccine education and messaging—and potentially identifies actionable targets for increasing *vaccine uptake*.

More broadly, the prediction of *vaccine uptake* may aid (1) vaccine supply chain and administration logistics by indicating areas that may need more or fewer vaccines, (2) targeted governmental messaging to locations with low predicted uptake, and (3) preparation of areas that may experience high cases of infection that could ultimately impact health care preparedness and infrastructure. The proposed method could also be applied to other mandated or government-recommended vaccines (eg, influenza and human papillomavirus) to facilitate the aforementioned logistics. Locally, *vaccine uptake* prediction could facilitate local messaging and prepare health care institutions for vaccine rollout and potential hospital overload. Nationally, prediction might inform public health officials and government communication bodies that are responsible for messaging and vaccine rollout with the goal of improving *vaccine uptake* and limiting infection and hospital overload.

## Methods

### Recruitment

Similar recruitment procedures for a smaller population-based study have been described previously [61-63]. In this study, participants were randomly sampled from the general US population using an email survey database used by Gold Research, Inc. Gold Research administered questionnaires in December 2021 using recruitment formats such as (1) customer databases from large companies that participate in revenue-sharing agreements, (2) social media, and (3) direct mail. Recruited participants followed a double opt-in consent procedure that included primary participation in the study as well as secondary use of anonymized, deidentified data (ie, all identifying information was removed by Gold Research before retrieval by the research group) in secondary analyses (refer to the *Ethical Considerations* section for more detail). During consent procedures, participants provided demographic information (eg, age, race, and sex) to ensure that the sampled participants adequately represented the US census at the time of the survey (December 2021). Respondents were also presented with repeated test questions to screen out those providing random and illogical responses or showing flatline or speeder behavior. Participants who provided such data were flagged, and their data were removed.

Because other components of the survey required an adequate sample of participants with mental health conditions, Gold Research oversampled 15% (60,000/400,000) of the sample for mental health conditions, and >400,000 respondents were contacted to complete the questionnaire. Gold Research estimated that, of the 400,000 participants, >300,000 (>75%) either did not respond or declined to participate. Of the remaining 25% (100,000/400,000) who clicked on the survey link, >50% (52,000/100,000) did not fully complete the questionnaire. Of the ≥48,000 participants who completed the survey (ie, ≥48,000/400,000, ≥12% of the initial pool of queried persons), those who did not clear data integrity assessments were omitted. Participants who met quality assurance procedures (refer to the following section) were selected, with a limit of 4000 to 4050 total participants.

Eligible participants were required to be aged between 18 and 70 years at the time of the survey, comprehend the English language, and have access to an electronic device (eg, laptop or smartphone).

### Ethical Considerations

All participants provided informed consent, which included their primary participation in the study as well as the secondary use of their anonymized, deidentified data (ie, all identifying information removed by Gold Research before retrieval by the research group) in secondary analyses. This study was approved by the Northwestern University institutional review board (approval STU00213665) for the initial project start and later by the University of Cincinnati institutional review board (approval 2023-0164) as some Northwestern University investigators moved to the University of Cincinnati. All study approvals were in accordance with the Declaration of Helsinki. All participants were compensated with US $10 for taking part. Detailed survey instructions have been published previously [61-63].

### Quality Assurance and Data Exclusion

Three additional quality assurance measures were used to flag nonadhering participants: (1) participants who indicated that they had ≥10 clinician-diagnosed illnesses (refer to Figure S1 in Multimedia Appendix 1 [18,33,36,64-68] for a list), (2) participants who showed minimal variance in the picture-rating task (ie, all pictures were rated the same or the ratings varied only by 1 point; refer to the *Picture-Rating Task* section), and (3) inconsistencies between educational level and years of education *and* participants who completed the questionnaire in <800 seconds.

**Figure 2:**
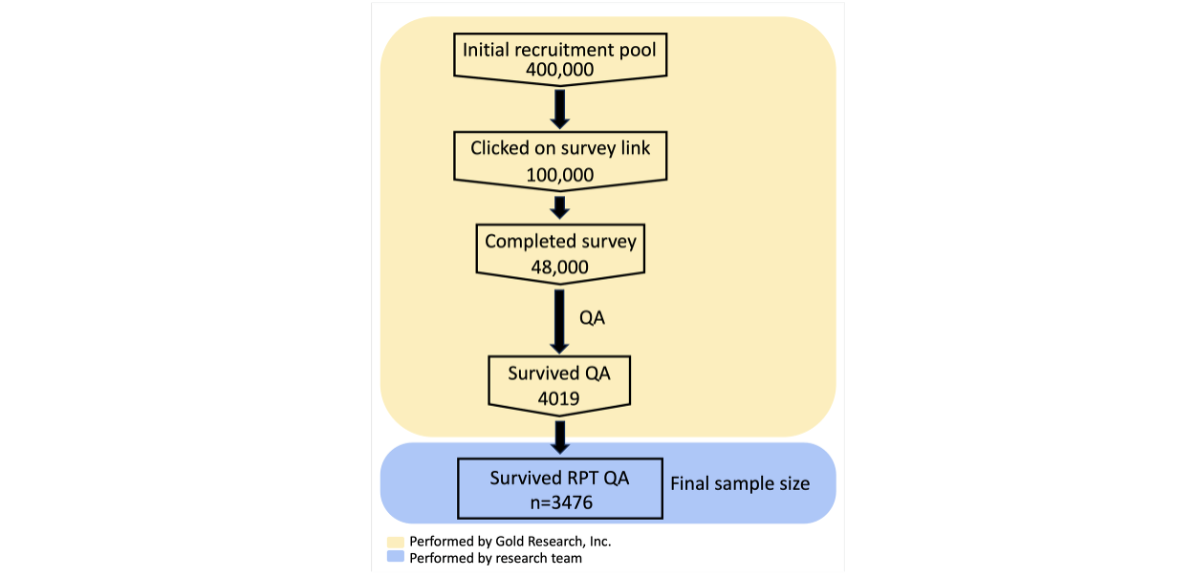
Participant exclusion flow diagram. Sample sizes are provided in each box. QA: quality assurance; RPT: relative preference theory.

Data from 4019 participants who passed the initial data integrity assessments were anonymized and then sent to the research team. Data were further excluded if the quantitative feature set derived from the picture-rating task was incomplete or if there were extreme outliers (refer to the *RPT Framework* section). Using these exclusion criteria, of the 4019 participants, 3476 (86.49%) were cleared for statistical analysis, representing 0.87% (3476/400,000) of the initial recruitment pool. A flowchart of participant exclusion is shown in Figure 2.

### Questionnaire

Participants were asked to report their age, sex, ethnicity, annual household income, marital status, employment status, and educational level. Participants were asked to report whether they had received the full vaccination (*yes* or *no* responses). At the time of the survey, participants were likely to have received either 2 doses of the Pfizer or Moderna vaccine or 1 dose of the Johnson & Johnson vaccine as per the Centers for Disease Control and Prevention guidelines. Participants were also asked to respond *yes* (they routinely followed the precaution) or *no* (they did not routinely follow the precaution) to 4 COVID-19 precaution behaviors: mask wearing, social distancing, washing or sanitizing hands, and not gathering in large groups (refer to Tables S1 and S2 in Multimedia Appendix 1 for the complete questions and sample sizes, respectively). In addition, participants completed a picture-rating task at 2 points during the survey (refer to the *Picture-Rating Task* section).

### Picture-Rating Task

A picture-rating task was administered to quantify participants’ degree of liking and disliking a validated picture set using pictures calibrated over large samples for their emotional intensity and valence [69,70]. Ratings from this task have been mathematically modeled using RPT to define graphical features of reward and aversion judgments. Each feature quantifies a core aspect of judgment, including risk aversion and loss aversion. Judgment variables have been shown to meet the criteria for lawfulness [37] that produce mechanistic models for prediction [33], with published relationships to brain circuitry [24-27,30] and psychiatric illness [28]. A more complete description of these judgment variables and their computation can be found in the *RPT Framework* section and in Table 1.

For this task, participants were shown 48 unique color images from the International Affective Picture System [69,70]. A total of 6 picture categories were used: sports, disasters, cute animals, aggressive animals, nature (beach vs mountains), and men and women dressed minimally, with 8 pictures per category (48 pictures in total; Figure 1A). These images have been used and validated in research on human emotion, attention, and preferences [69,70]. The images were displayed on the participants’ digital devices with a maximum size of 1204 × 768 pixels. Below each picture was a rating scale from −3 (*dislike very much*) to +3 (*like very much*), where 0 indicated indifference (Figure 1A). While there was no time limit for selecting a picture rating, participants were asked to rate the images as quickly as possible and use their first impression. Once a rating was selected, the next image was displayed.

**Table 1:** Judgment variable descriptions. Function and curve for derivation indicates which function space was used to derive each judgment variable (refer to the work by Markowitz [39,71]; equations are also discussed in the study by Kim et al [18]).

Judgment variable	Function and curve for derivation	Description
Loss aversion	Value function and (K,H) curve	The degree to which one overweighs negative stimuli against positive stimuli
Risk aversion	Value function and (K,H) curve	The degree to which one prefers an uncertain high-value outcome to something certain but of a lower value
Loss resilience	Value function and (K,H) curve	Measures one’s preference to accept a certain loss over an uncertain loss. It is similar to risk aversion but in the domain of losses.
Ante	Value function and (K,H) curve	What one is willing to pay to enter a game of chance (eg, poker)
Insurance	Value function and (K,H) curve	The amount of security one is willing to acquire to avoid negative outcomes
Peak positive risk	Limit function and (K, *σ*) curve	Per the decision utility equation by Markowitz [39,71], this is the peak risk regarding approach choices that must be overcome for approach behavior to occur.
Peak negative risk	Limit function and (K, *σ*) curve	Per the decision utility equation by Markowitz [39,71], this is the peak risk regarding avoidance choices that must be overcome for avoidance behavior to occur.
Reward tipping point	Limit function and (K, *σ*) curve	Per the decision utility equation by Markowitz [39,71], this is the reward value beyond which approach choices are made.
Aversion tipping point	Limit function and (K, *σ*) curve	Per the decision utility equation by Markowitz [39,71], this is the intensity of aversion beyond which avoidance choices are made.
Total reward risk	Limit function and (K, *σ*) curve	Total value of reward across the range of risks associated with those positive outcomes
Total aversion risk	Limit function and (K, *σ*) curve	The total amount of aversion across the range of risks associated with those negative outcomes
Reward-aversion trade-off	Value function and (H_+_,H_−_) curve	This angle represents the average bias of information toward approach or avoidance behavior.
Trade-off range	Value function and (H_+_,H_−_) curve	The variance or bias toward approach versus avoidance behavior and one metric of the range in a person’s portfolio of preference
Reward-aversion consistency	Value function and (H_+_,H_−_) curve	A continuum between how much an individual has conflict versus indifference in their reward-aversion preference—where conflict means that they both like and dislike something and indifference means that they do not like or dislike something
Consistency range	Value function and (H_+_,H_−_) curve	How much a person swings between conflict and indifference in their preferences; it is a second metric regarding the range in a person’s portfolio of preference

### RPT Framework

Ratings from the picture-rating task were analyzed using an RPT framework. This framework fits approach and avoidance curves and derives mathematical features from graphical plots (Figures 1B-1D). These methods have been described at length in prior work and are briefly described in this section [11,18,33,36]. More complete descriptions and quality assurance procedures can be found in Multimedia Appendix 1.

At least 15 judgment variables can be mathematically derived from this framework and are psychologically interpretable; they have been validated using both operant keypress [9,25-27] and picture-rating tasks [11,34]. The 15 judgment variables are loss aversion, risk aversion, loss resilience, ante, insurance, peak positive risk, peak negative risk, reward tipping point, aversion tipping point, total reward risk, total aversion risk, reward-aversion trade-off, trade-off range, reward-aversion consistency, and consistency range. Loss aversion, risk aversion, loss resilience, ante, and insurance are derived from the logarithmic or power-law fit of mean picture ratings (*K*) versus entropy of ratings (*H*); this is referred to as the value function (Figure 1B). Peak positive risk, peak negative risk, reward tipping point, aversion tipping point, total reward risk, and total aversion risk are derived from the quadratic fit of *K* versus the SD of picture ratings (*σ*); this is referred to as the limit function (Figure 1C). Risk aversion trade-off, trade-off range, risk aversion consistency, and consistency range are derived from the radial fit of the pattern of avoidance judgments (*H_−_*) versus the pattern of approach judgments (*H_+_*); this is referred to as the trade-off function (Figure 1D). Value (Figure S2A in Multimedia Appendix 1), limit (Figure S2B in Multimedia Appendix 1), and trade-off (Figure S2C in Multimedia Appendix 1) functions were plotted for 500 randomly sampled participants, and nonlinear curve fits were assessed for goodness of fit, yielding *R*^2^, adjusted *R*^2^, and the associated *F* statistic for all participants (Figure S2D in Multimedia Appendix 1). Only the logarithmic and quadratic fits are listed in Table S3 in Multimedia Appendix 1. Each feature describes a quantitative component of a participant’s reward and aversion judgment (refer to Table 1 for abbreviated descriptions and Multimedia Appendix 1 for complete descriptions). Collectively, the 15 RPT features will be henceforth referred to as “judgment variables.” The summary statistics for these variables can be found in Table S3 in Multimedia Appendix 1.

### Statistical and Machine Learning Analyses

#### Overview

Wilcoxon rank sum tests, chi-square tests, and Gini importance plotting were performed in Stata (version 17; StataCorp) [72]. Machine learning algorithms were run in Python (version 3.9; Python Software Foundation) [73], where the scikit-learn (version 1.2.2) [74] and imbalanced-learn (version 0.10.1) [75] libraries were used. Post hoc mediation and moderation analyses were performed in R (version 4.2.0; R Foundation for Statistical Computing) [76].

#### Demographic and Judgment Variable Differences by Vaccination Uptake

Each of the 7 demographic variables (age, income, marital status, employment status, ethnicity, educational level, and sex) was assessed for differences using *yes* or *no* responses to receiving the full COVID-19 vaccination (2525/3476, 72.64% *yes* responses and 951/3476, 27.36% *no* responses), henceforth referred to as *vaccine uptake*. Ordinal (income and educational level) and continuous (age) demographic variables were analyzed using the Wilcoxon rank sum test (*α*=.05). Expected and actual rank sums were reported using Wilcoxon rank sum tests. Nominal variables were analyzed using the chi-square test (*α*=.05). For significant chi-square results, demographic response percentages were computed to compare the fully vaccinated and not fully vaccinated groups.

Each of the 15 judgment variables was assessed for differences across *yes* or *no* responses to *vaccine uptake* using the Wilcoxon rank sum test (*α*=.05). The expected and actual rank sums were reported. Significant results (*α*<.05) were corrected for multiple comparisons using the Benjamini-Hochberg correction, and *Q* values of <0.05 (*Q*_Hoch_) were reported.

#### Prediction Analyses

Logistic regression, random forest, and BRF were used to predict *vaccine uptake* using judgment, demographic, and COVID-19 precaution variables. Gini plots were produced for random forest and BRF to determine the importance of the judgment variables in predicting COVID-19 vaccination. The BRF algorithm balances the samples by randomly downsampling the majority class at each bootstrapped iteration to match the number of samples in the minority class. To provide greater certainty about the results, random forest and logistic regression were performed to compare with BRF results.

Two sets of BRF, random forest, and logistic regression analyses were run: (1) with the 7 demographic variables and 15 judgment variables included as predictors and (2) with the 7 demographic variables, 15 judgment variables, and 4 COVID-19 precaution behaviors included as predictors. COVID-19 precaution behaviors included *yes* or *no* responses to wearing a mask, social distancing, washing hands, and avoiding large gatherings (refer to Table S1 in Multimedia Appendix 1 for more details). The sample sizes for *yes* or *no* responses to the COVID-19 precaution behavior questions are provided in Table S2 in Multimedia Appendix 1. For all 3 models, 10-fold cross-validation was repeated 100 times to obtain performance metrics, where data were split for training (90%) and testing (10%) for each of the 10 iterations in cross-validation. The averages of the performance metrics were reported across 100 repeats of 10-fold cross-validation for the test sets. The reported metrics included accuracy, recall, specificity, negative predictive value (NPV), precision, and area under the receiver operating characteristic curve (AUROC). For BRF, the Python toolbox *imbalanced-learn* was used to build the classifier, where the training set for each iteration of cross-validation was downsampled but the testing set was unchanged (ie, imbalanced). That is, downsampling *only* occurred with the bootstrapped samples for training the model, and balancing was not performed on the testing set. The default number of estimators was 100, and the default number of tree splits was 10; the splits were created using the Gini criterion. In separate analyses, estimators were increased to 300, and splits were increased to 15 to test model performance. Using the scikit-learn library, the same procedures used for BRF were followed for random forest *without* downsampling. Logistic regression without downsampling was implemented with a maximum of 100 iterations and optimization using a limited-memory Broyden-Fletcher-Goldfarb-Shanno solver. For logistic regression, model coefficients with respective SEs, *z* statistics, *P* values, and 95% CIs were reported.

Relative feature importance based on the Gini criterion (henceforth referred to as *Gini importance*) was determined from BRF and random forest using the .feature_importances_ attribute from scikit-learn, and results were reported as the mean decrease in the Gini score and plotted in Stata. To test model performance using only the top predictors, two additional sets of BRF analyses were run: (1) with the top 3 features as predictors and (2) with the top 3 features and 15 judgment variables as predictors.

#### Post Hoc Mediation and Moderation

Given the importance of both judgment variables and demographic variables (refer to the *Results* section), we evaluated post hoc how age, income, and educational level (ie, the top 3 predictors) might statistically influence the relationship between the 15 judgment variables and COVID-19 *vaccine uptake*. To identify statistical mechanisms influencing our prediction results, we used mediation and moderation, which can (1) determine the directionality between variables and (2) assess variable influence in statistical relationships. Mediation is used to determine whether one variable, the mediator, statistically improves the relationship between 2 other variables (independent variables [IVs] and dependent variables [DVs]) [77-80]. When mediating variables improve a relationship, the mediator is said to sit in the statistical pathway between the IVs and DVs [77,80,81]. Moderation is used to test whether the interaction between an IV and a moderating variable predicts a DV [81,82].

For mediation, primary and secondary mediations were performed. Primary mediations included each of the 15 judgment behaviors as the IV, each of the 3 demographic variables (age, income, and educational level) as the mediator, and *vaccine uptake* as the DV. Secondary mediations held the 15 judgment behaviors as the mediator, the 3 demographic variables as the IV, and *vaccine uptake* as the DV. For moderation, the moderating variable was each of the 3 demographic variables (age, income, and educational level), the IV was each of the 15 judgment behaviors, and the DV was *vaccine uptake*. The mathematical procedures for mediation and moderation can be found in Multimedia Appendix 1.

## Results

### Demographic Assessment

Of the 400,000 persons queried by Gold Research, Inc, 48,000 (12%) completed the survey, and 3476 (0.87%) survived all quality assurance procedures. Participants were predominately female, married, and White individuals; employed full time with some college education; and middle-aged (mean age 51.40, SD 14.92 years; Table 2). Of the 3476 participants, 2525 (72.64%) reported receiving a full dose of a COVID-19 vaccine, and 951 (27.36%) reported not receiving a full dose. Participants who indicated full vaccination were predominately female, married, White individuals, and retired; had some college education; and were older on average (mean age 54.19, SD 14.13 years) when compared to the total cohort. Participants who indicated that they did not receive the full vaccine were also predominately female, married, and White individuals. In contrast to those who received the full vaccination, those not fully vaccinated were predominately employed full time, high school graduates, and of average age (mean age 43.98, SD 14.45 years; median age 45, IQR 32-56 years) when compared to the total cohort. Table 2 summarizes the demographic group sample size percentages for the total cohort, those fully vaccinated, and those not fully vaccinated.

When comparing percentages between vaccination groups, a higher percentage of male individuals were fully vaccinated, and a higher percentage of female individuals were not fully vaccinated (Table 2). In addition, a higher percentage of married, White and Asian or Pacific Islander, and retired individuals indicated receiving the full vaccine when compared to the percentages of those who did not receive the vaccine (Table 2). Conversely, a higher percentage of single, African American, and unemployed individuals indicated *not* receiving the full vaccine (Table 2).

**Table 2:** Demographic summary of the sample (N=3476).

Demographics		Vaccine uptake			Total
		Fully vaccinated (n=2525)	Not fully vaccinated (n=951)		
Age (y), median (IQR)		59 (44-46)	45 (32-56)	55 (39-65)	
**Income level (US $), n (%)**					
	<25,000	338 (13.4)	288 (30.3)	626 (18)	
	25,000-50,000	621 (24.6)	285 (30)	906 (26.1)	
	50,000-75,000	500 (19.8)	194 (20.4)	694 (20)	
	75,000-100,000	428 (17)	98 (10.3)	526 (15.1)	
	100,000-150,000	388 (15.4)	57 (6)	445 (12.8)	
	150,000-300,000	204 (8.1)	26 (2.7)	230 (6.6)	
	>300,000	46 (1.8)	3 (0.3)	49 (1.4)	
**Sex, n (%)**					
	Male	1038 (41.1)	286 (30.1)	1324 (38.1)	
	Female	1478 (58.5)	660 (69.4)	2138 (61.5)	
	Prefer not to answer	9 (0.4)	5 (0.5)	14 (0.4)	
**Race and ethnicity, n (%)**					
	Asian or Pacific Islander	109 (4.3)	13 (1.4)	122 (3.5)	
	Black or African American	126 (5)	101 (10.6)	227 (6.5)	
	Hispanic	89 (3.5)	34 (3.6)	123 (3.5)	
	Native American or Alaska Native	11 (0.4)	15 (1.6)	26 (0.7)	
	White	2152 (85.2)	756 (79.5)	2908 (83.7)	
	Mixed	15 (0.6)	15 (1.6)	30 (0.9)	
	Other	9 (0.4)	7 (0.7)	16 (0.5)	
	Prefer not to answer	14 (0.6)	10 (1.1)	24 (0.7)	
**Marital status, n (%)**					
	Single	478 (18.9)	288 (30.3)	766 (22)	
	Married	1426 (56.5)	362 (38.1)	1788 (51.4)	
	Divorced	296 (11.7)	120 (12.6)	416 (12)	
	Separated	36 (1.4)	28 (2.9)	64 (1.8)	
	Widowed	129 (5.1)	23 (2.4)	152 (4.4)	
	Living with partner	144 (5.7)	125 (13.1)	269 (7.7)	
	Other or prefer not to answer	16 (0.6)	5 (0.5)	21 (0.6)	
**Employment status, n (%)**					
	Unemployed	254 (10.1)	227 (23.9)	481 (13.8)	
	Employed full time	906 (35.9)	338 (35.5)	1244 (35.8)	
	Employed part time	226 (9)	86 (9)	312 (9)	
	Self-employed	97 (3.8)	68 (7.2)	165 (4.7)	
	>1 job	3 (0.1)	4 (0.4)	7 (0.2)	
	Retired	931 (36.9)	148 (15.6)	1079 (31)	
	Prefer not to answer	108 (4.3)	80 (8.4)	188 (5.4)	
**Educational level, n (%)**					
	Some high school	44 (1.7)	57 (6)	101 (2.9)	
	High school graduate	410 (16.2)	324 (34.1)	734 (21.1)	
	Some college	710 (28.1)	320 (33.6)	1030 (29.6)	
	Bachelor’s degree	652 (25.8)	125 (13.1)	777 (22.4)	
	Some graduate school	142 (5.6)	34 (3.6)	176 (5.1)	
	Graduate school graduate	488 (19.3)	76 (8)	564 (16.2)	
	Postgraduate or doctorate	79 (3.1)	15 (1.6)	94 (2.7)	

### Analysis of Machine Learning Features

#### Demographic Variable Differences by Vaccine Uptake

Age, income level, and educational level significantly differed between those who did and did not receive the vaccine (Wilcoxon rank sum test *α*<.05; Table 3). Those who indicated full vaccination were, on average, older (median age 59 y), had a higher annual household income (median reported income level US $50,000-$75,000), and had higher levels of education (the median reported educational level was a bachelor’s degree).

Chi-square tests revealed that marital status, employment status, sex, and ethnicity also varied by full *vaccine uptake* (chi-square *α*<.05; Table 3).

**Table 3:** Wilcoxon rank sum and chi-square test results (*α*=.05). Expected and actual rank sums for each vaccine uptake group are reported. “Higher” indicates that the actual rank sum was higher than expected, and “lower” indicates that the actual rank sum was lower than expected.

Demographic variable and full vaccination status		Rank sum	Expected	Higher or lower	Test		*P* value	
**Age**						Rank sum		<.001
	Yes	4,873,514.5	4,389,712.5	Higher				
	No	1,169,511.5	1,653,313.5	Lower				
**Income**						Rank sum		<.001
	Yes	4,770,430.5	4,389,712.5	Higher				
	No	1,272,595.5	1,653,313.5	Lower				
**Marital status**						Chi-square		<.001
	N/A^a^	N/A	N/A	N/A				
**Employment status**						Chi-square		<.001
	N/A	N/A	N/A	N/A				
**Ethnicity**						Chi-square		<.001
	N/A	N/A	N/A	N/A	Chi-square		<.001	
**Educational level**						Rank sum		<.001
	Yes	4,794,497.5	4,389,712.5	Higher				
	No	1,248,528.5	1,653,313.5	Lower				
**Sex**						Chi-square		<.001
	N/A	N/A	N/A	N/A				

^a^N/A: not applicable.

#### Judgment Variable Differences by Vaccine Uptake

In total, 10 of the 15 judgment variables showed nominal rank differences (*α*<.05), and 9 showed significant rank differences after correction for multiple comparisons (*Q*_Hoch_<0.05) between those who indicated full vaccination and those who indicated that they did not receive the full vaccination (Table 4). The 10 features included loss aversion, risk aversion, loss resilience, ante, insurance, peak positive risk, peak negative risk, total reward risk, total aversion risk, and trade-off range. Those who indicated full vaccination exhibited lower loss aversion, ante, peak positive risk, peak negative risk, total reward risk, and total aversion risk as well as higher risk aversion, loss resilience, insurance, and trade-off range when compared to the expected rank sum. Those who did not receive the full vaccination exhibited lower risk aversion, loss resilience, insurance, and trade-off range and higher loss aversion, ante, peak positive risk, peak negative risk, total reward risk, and total aversion risk when compared to the expected rank sum.

**Table 4:** Judgment variable differences by vaccine uptake (yes vs no responses to the full vaccination question). Expected and actual rank sums for each vaccine uptake group are reported from Wilcoxon rank sum tests (*α*=.05). “Higher” indicates that the actual rank sum was higher than expected, and “lower” indicates that the actual rank sum was lower than expected.

Judgment variable and full vaccination uptake			Rank sum		Expected		Higher or lower		*P* value			*Q*_Hoch_	
**Loss aversion**										.02			0.10
	No	1,716,061.5		1,653,313.5		Higher							
	Yes	4,326,964.5		4,389,712.5		Lower							
**Risk aversion**										<.001			0.009
	No	1,551,369.5		1,653,313.5		Lower							
	Yes	4,491,656.5		4,389,712.5		Higher							
**Loss resilience**										<.001			<0.001
	No	1,524,220		1,653,313.5		Lower							
	Yes	4,518,806		4,389,712.5		Higher							
**Ante**										<.001			<0.001
	No	1,803,995		1,653,313.5		Higher							
	Yes	4,239,031		4,389,712.5		Lower							
**Insurance**										<.001			<0.001
	No	1,535,572.5		1,653,313.5		Lower							
	Yes	4,507,453.5		4,389,712.5		Higher							
**Peak positive risk**										<.001			<0.001
	No	1,801,272		1,653,313.5		Higher							
	Yes	4,241,754		4,389,712.5		Lower							
**Peak negative risk**										.001			0.01
	No	1,738,603		1,653,313.5		Higher							
	Yes	4,304,423		4,389,712.5		Lower							
**Reward tipping point**										.30			N/A^a^
	No	1,625,945		1,653,313.5		N/A							
	Yes	4,417,081		4,389,712.5		N/A							
**Aversion tipping point**										.94			N/A
	No	1,651,477		1,653,313.5		N/A							
	Yes	4,391,549		4,389,712.5		N/A							
**Total reward risk**										<.001			<0.001
	No	1,771,478		1,653,313.5		Higher							
	Yes	4,271,548		4,389,712.5		Lower							
**Total aversion risk**										.002			0.01
	No	1,737,001.5		1,653,313.5		Higher							
	Yes	4,306,024.5		4,389,712.5		Lower							
**Reward-aversion trade-off**										.31			N/A
	No	1,626,357		1,653,313.5		N/A							
	Yes	4,416,669		4,389,712.5		N/A							
**Trade-off range**										<.001			<0.001
	No	1,529,559.5		1,653,313.5		Lower							
	Yes	4,513,466.5		4,389,712.5		Higher							
**Reward-aversion consistency**										.20			N/A
	No	1,619,169.5		1,653,313.5		N/A							
	Yes	4,423,856.5		4,389,712.5		N/A							
**Consistency range**										.22			N/A
	No	1,620,913		1,653,313.5		N/A							
	Yes	4,422,113		4,389,712.5		N/A							

^a^N/A: not applicable.

### Machine Learning Results: Predicting Vaccination Uptake

#### Prediction Results

With the inclusion of demographic and judgment variables, the BRF classifier with the highest accuracy (68.9%) and precision (86.7%) in predicting *vaccine uptake* resulted when the number of estimators was set to 300 and the number of splits was set to 10 (Table 5). With the addition of 4 COVID-19 precaution behaviors, the BRF classifier with the highest accuracy (70.8%) and precision (87.8%) to predict *vaccine uptake* occurred when the number of estimators was set to 300 and the number of splits was set to 10. It is notable that specificity was consistently >72%, precision was >86%, and the AUROC was >75% but the NPV was consistently <50%. For random forest and logistic regression, recall and accuracy values were higher than those for BRF, but specificity was always <39%, indicating a lower performance in predicting those who did not receive the vaccine. Precision was also lower, yet the AUROC was consistent with that of the BRF results.

**Table 5:** Predicting COVID-19 vaccine uptake using three machine learning algorithms^a^.

Method and features		Splits	Estimators	Accuracy (%)	Recall (%)	Specificity (%)	Precision (%)	NPV^b^ (%)	AUROC^c^ (%)
**BRF^d^**									
	Judgment+demographic	10	100	68.4	67.1	72.1	86.5	45.2	75.2
	Judgment+demographic	15	100	68.3	66.9	72.2	86.5	45.2	75.1
	Judgment+demographic	10	300	68.9	67.5	72.4	86.7	45.7	75.6
	Judgment+demographic	15	300	68.8	67.5	72.3	86.7	45.7	75.6
	Judgment+demographic+covid_beh	10	100	70.6	69.2	74.2	87.7	47.6	78.6
	Judgment+demographic+covid_beh	15	100	70.4	69.0	74.2	87.7	47.6	78.6
	Judgment+demographic+covid_beh	10	300	70.8	69.6	74.2	87.8	48.0	79.0
	Judgment+demographic+covid_beh	15	300	70.8	69.5	74.2	87.8	48.0	79.0
**Random forest**									
	Judgment+demographic	10	100	74.9	91.6	30.0	77.7	57.7	74.7
	Judgment+demographic	15	100	74.8	91.7	30.1	77.7	57.9	74.7
	Judgment+demographic	10	300	75.0	92.3	29.1	77.6	58.8	75.1
	Judgment+demographic	15	300	75.0	92.3	29.0	77.6	59.0	75.1
	Judgment+demographic+covid_beh	10	100	77.0	91.7	38.1	79.8	63.6	78.1
	Judgment+demographic+covid_beh	15	100	77.0	91.6	38.3	79.8	63.6	78.1
	Judgment+demographic+covid_beh	10	300	77.3	92.1	37.9	79.7	64.8	78.5
	Judgment+demographic+covid_beh	15	300	77.3	92.1	38.0	79.8	64.8	78.5
**Logistic regression**									
	Judgment+demographic	N/A^e^	N/A	74.8	91.6	30.2	77.7	57.8	75.5
	Judgment+demographic+covid_beh	N/A	N/A	77.0	92.1	37.2	79.6	64.0	79.1

^a^A total of 15 judgment variables (Table 4), 7 demographic variables (Table 3), and 4 COVID-19 precaution behavior (covid_beh) variables (Table S1 in Multimedia Appendix 1) were included in balanced random forest, random forest, and logistic regression models to predict COVID-19 *vaccine uptake*. We used 10-fold cross-validation, where the data were split 90-10 for each of the 10 iterations.

^b^NPV: negative predictive value.

^c^AUROC: area under the receiver operating characteristic curve.

^d^BRF: balanced random forest.

^e^N/A: not applicable.

#### Feature Importance for BRF and Random Forest

**Figure 3:**
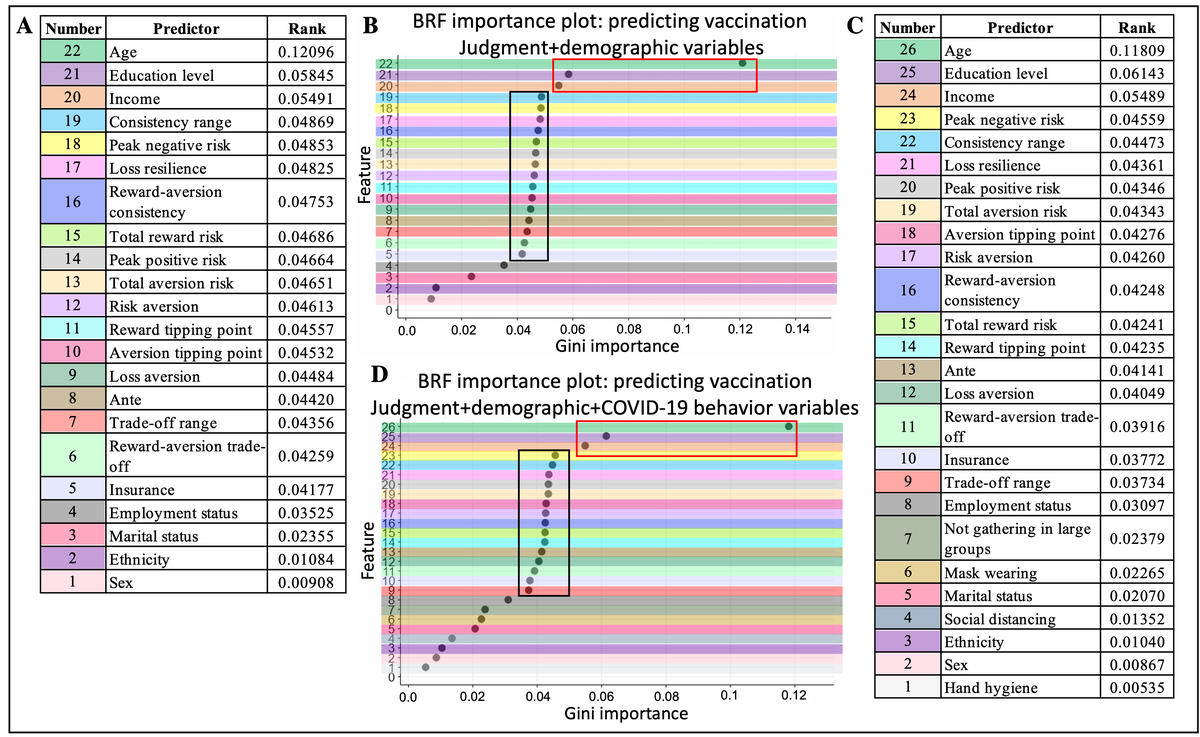
Balanced random forest (BRF) Gini importance plots. (A) Table of predictor ranks for the model including 15 judgment and 7 demographic variables. (B) Gini plot of the ranks in (A); the red box outlines the top 3 features (age, income, and educational level), and the black box outlines the 15 judgment variables. (C) Table of predictor ranks for the model including 15 judgment, 7 demographic, and 4 COVID-19 precaution behavior variables. (D) Gini plot of the ranks in (C); the red box outlines the top 3 features (age, income, and educational level), and the black box outlines the 15 judgment variables.

Regarding BRF, Gini importance was highest for age, educational level, and income in both BRF classifiers (both without [Figures 3A and 3B] and with [Figures 3C and 3D] inclusion of the COVID-19 precaution behaviors; refer to the clusters outlined in red in Figures 3B and 3D). For both BRF classifiers, the top 3 predictors (age, income, and educational level) had a combined effect of 23.4% on the Gini importance for prediction. Following these predictors, the 15 judgment variables had similar importance scores for both BRF classifiers (range 0.037-0.049; refer to the clusters outlined in black in Figures 3B and 3D). These 15 predictors had a combined effect of 62.9% to 68.7% on the Gini importance for prediction, indicating that judgment variables were *collectively* the most important for prediction outcomes. The least important features for predicting vaccination status were demographic variables regarding employment status, marital status, ethnicity, sex, and the 4 COVID-19 precaution behaviors. These predictors only contributed 7.3% to the Gini importance for prediction. As a follow-up analysis, BRF analyses were run using the top 3 features from both the Gini importance plots (age, educational level, and income; Table S4 in Multimedia Appendix 1) and the top 3 features plus 15 judgment variables (Table S5 in Multimedia Appendix 1). The results did not outperform those presented in Table 5.

**Figure 4:**
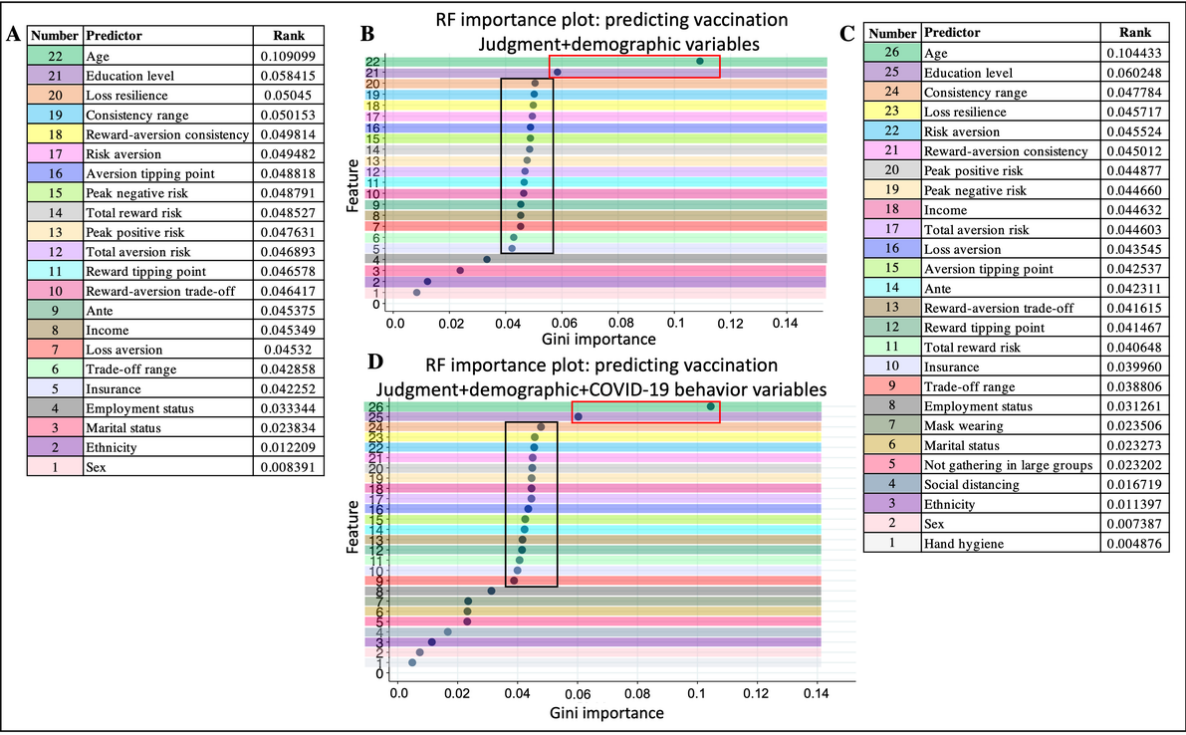
Random forest (RF) Gini importance plots. (A) Table of predictor ranks for the model including 15 judgment and 7 demographic variables. (B) Gini plot of the ranks in (A); the red box outlines the top 2 features (age and educational level), and the black box outlines the 15 judgment variables. (C) Table of predictor ranks for the model including 15 judgment, 7 demographic, and 4 COVID-19 precaution behavior variables. (D) Gini plot of the ranks in (C); the red box outlines the top 2 features (age and educational level), and the black box outlines the 15 judgment variables.

For random forest, the Gini importance was highest for age and educational level (Figure 4). These top 2 predictors had a combined effect of 16.5% to 16.8% for the 2 models (Figures 4A and 4C). Following these predictors, the 15 judgment variables and the income variable had similar Gini importance, with a combined effect of 69.4% to 75.5% for Gini importance. The least important predictors mirrored those of the BRF results.

#### Logistic Regression Model Statistics

Both model 1 (demographic and judgment variables) and model 2 (demographic, judgment, and COVID-19 precaution behavior variables) were significant (*P*<.001). The model statistics are provided in Tables 6 (model 1) and 7 (model 2). In model 1, age, income, marital status, employment status, sex, educational level, ante, aversion tipping point, reward-aversion consistency, and consistency range were significant (*α*<.05). In model 2, age, income, marital status, employment status, sex, educational level, risk aversion, ante, peak negative risk, mask wearing, and not gathering in large groups were significant (*α*<.05).

**Table 6:** Logistic regression model 1 results (demographic and judgment variables predict vaccine uptake)^a^.

Variable	Variable coefficient	*P* value	*z* statistic (SE; 95% CI)
Age	0.039	<.001	11.70 (0.003; 0.033 to 0.046)
Income	0.262	<.001	7.91 (0.033; 0.197 to 0.326)
Marital status	−0.061	.03	−2.21 (0.028; −0.116 to −0.007)
Employment status	0.048	.04	2.03 (0.023; 0.002 to 0.093)
Sex	−0.244	.008	−2.65 (0.092; −0.425 to −0.064)
Ethnicity	0.017	.66	0.44 (0.038; −0.057 to 0.091)
Educational level	0.232	<.001	6.84 (0.034; 0.166 to 0.299)
Loss aversion	0.002	.78	0.28 (0.009; −0.015 to 0.019)
Risk aversion	−0.605	.06	−1.89 (0.320; −1.232 to 0.023)
Loss resilience	0.353	.13	1.51 (0.235; −0.107 to 0.813)
Ante	−1.079	.04	−2.08 (0.519; −2.096 to −0.061)
Insurance	0.364	.45	0.75 (0.483; −0.582 to 1.311)
Peak positive risk	−0.176	.40	−0.85 (0.208; −0.584 to 0.231)
Peak negative risk	−0.383	.05	−1.93 (0.199; −0.773 to 0.006)
Reward tipping point	0.126	.26	1.14 (0.110; −0.090 to 0.342)
Aversion tipping point	−0.499	.03	−2.19 (0.228; −0.947 to −0.052)
Total reward risk	−0.020	.52	−0.64 (0.032; −0.083 to 0.042)
Total aversion risk	0.114	.09	1.76 (0.065; −0.013 to 0.240)
Reward-aversion trade-off	−0.002	.63	−0.49 (0.004; −0.009 to 0.006)
Trade-off range	0.012	.12	1.54 (0.008; −0.002 to 0.028)
Reward-aversion consistency	−0.367	.047	−1.98 (0.185; −0.730 to −0.004)
Consistency range	−0.399	.04	−2.06 (0.194; −0.779 to −0019)

^a^Overall model: *P*<.001; pseudo-*R*^2^=0.149; log-likelihood=−1736.8; log-likelihood null=−2039.7.

**Table 7:** Logistic regression model 2 results (demographic, judgment, and COVID-19 precaution variables predict vaccine uptake)^a^.

Variable	Variable coefficient	*P* value	*z* statistic (SE; 95% CI)
Age	0.040	<.001	11.33 (0.003; 0.033 to 0.046)
Income	0.310	<.001	8.82 (0.035; 0.214 to 0.379)
Marital status	−0.064	.03	−2.18 (0.029; −0.121 to −0.006)
Employment status	0.049	.04	2.02 (0.024; 0.002 to 0.097)
Sex	−0.244	.01	−2.55 (0.096; −0.432 to −0.056)
Ethnicity	0.012	.75	0.32 (0.039; −0.063 to 0.088)
Educational level	0.264	<.001	7.42 (0.036; 0.194 to 0.333)
Loss aversion	0.008	.39	0.87 (0.009; −0.010 to 0.026)
Risk aversion	−0.663	.045	−2.00 (0.331; −1.311 to −0.014)
Loss resilience	0.216	.37	0.89 (0.243; −0.259 to 0.691)
Ante	−1.207	.03	−2.23 (0.542; −2.270 to −0.144)
Insurance	0.422	.40	0.84 (0.503; 0.564 to 1.408)
Peak positive risk	0.079	.72	0.36 (0.220; −0.353 to 0.511)
Peak negative risk	−0.434	.04	−2.09 (0.208; −0.842 to −0.026)
Reward tipping point	0.166	.22	1.24 (0.134; −0.097 to 0.430)
Aversion tipping point	−0.412	.08	−1.74 (0.237; −0.876 to 0.052)
Total reward risk	−0.044	.26	−1.12 (0.039; −0.121 to 0.033)
Total aversion risk	0.107	.12	1.58 (0.068; −0.026 to 0.240)
Reward-aversion trade-off	−0.001	.78	−0.28 (0.004; −0.009 to 0.007)
Trade-off range	0.014	.09	1.68 (0.008; −0.002 to 0.029)
Reward-aversion consistency	0.254	.20	1.29 (0.197; −0.133 to 0.641)
Consistency range	0.064	.76	0.31 (0.206; −0.340 to 0.468)
Mask wearing	−0.875	<.001	−5.31 (0.165; −1.198 to −0.552)
Social distancing	−0.384	.06	−1.92 (0.201; −0.778 to 0.009)
Hand hygiene	−0.155	.44	−0.78 (0.198; −0.543 to 0.234)
Not gathering in large groups	−0.680	<.001	−4.31 (0.158; −0.990 to −0.371)

^a^Overall model: *P*<.001; pseudo-*R*^2^=0.206; log-likelihood=−1620.0; log-likelihood null=−2039.7.

### Post Hoc Mediation and Moderation

Because judgment variables and demographic variables (age, income, and educational level) were important predictors, we evaluated post hoc whether demographics statistically mediated or moderated the relationship between each of the 15 judgment variables and binary responses to COVID-19 vaccination.

For primary mediations, age significantly mediated the statistical relationship between 11 judgment variables and *vaccine uptake* (*α*<.05; Table 8), income mediated 8 relationships *α*< <.05; Table 8), and educational level mediated 9 relationships (*α*<.05; Table 8). In total, 7 judgment variables overlapped across the 3 models: loss resilience, ante, insurance, peak positive risk, peak negative risk, risk aversion trade-off, and consistency range. Of these, 5 significantly differed between *vaccine uptake* (those fully vaccinated and those not): loss resilience, ante, insurance, peak positive risk, and peak negative risk (Table 3). Thus, 2 judgment features did not differ by *vaccine uptake* but were connected with uptake by significant mediation.

For the secondary mediation analyses, 5 judgment variables mediated the statistical relationship between age and *vaccine uptake*; these variables overlapped with the 11 findings of the primary mediation analyses. Furthermore, 4 judgment variables mediated the statistical relationship between income and *vaccine uptake*; these variables overlapped with the 8 findings of the primary mediation analyses. Finally, 4 judgment variables mediated the statistical relationship between educational level and *vaccine uptake*; these variables overlapped with the 9 findings of the primary mediation analyses. In all secondary analyses, approximately half of the judgment variables were involved in mediation as compared to the doubling of judgment variable numbers observed in the primary mediation analyses. In the secondary mediation analyses, the same 4 judgment variables were found in both primary and secondary mediation results, indicating a mixed mediation framework.

**Table 8:** Mediation and moderation results (*α*=.05). Mediator and moderator variables appear in boldface.

Independent variable				*P* value
**Mediator**				
	**Age**			
		Risk aversion	<.001	
		Loss resilience	<.001	
		Ante	<.001	
		Insurance	.003	
		Peak positive risk	<.001	
		Peak negative risk	.004	
		Aversion tipping point	.004	
		Total reward risk	.01	
		Risk aversion trade-off	.03	
		Trade-off range	<.001	
		Consistency range	<.001	
	**Income**			
		Loss resilience	.005	
		Ante	.002	
		Insurance	<.001	
		Peak positive risk	<.001	
		Peak negative risk	.02	
		Risk aversion trade-off	<.001	
		Risk aversion consistency	<.001	
		Consistency range	.007	
	**Education**			
		Risk aversion	.03	
		Loss resilience	.009	
		Ante	<.001	
		Insurance	<.001	
		Peak positive risk	<.001	
		Peak negative risk	.009	
		Risk aversion trade-off	.004	
		Risk aversion consistency	<.001	
		Consistency range	<.001	
	**Loss resilience**			
		Age	.02	
		Income	.02	
		Education	.03	
	**Ante**			
		Age	<.001	
		Income	.008	
		Education	<.001	
	**Insurance**			
		Age	.02	
		Income	.01	
		Education	.01	
	**Peak positive risk**			
		Age	.002	
		Income	.002	
	**Peak negative risk**			
		Age	.04	
		Education	.001	
**Moderator**				
	**Age**			
		Risk aversion trade-off	<.001	
	**Income**			
		Loss resilience	<.001	

From the moderation analyses, only 2 interactions out of a potential 45 were observed. Age interacted with risk aversion trade-off, and income interacted with loss resilience to statistically predict *vaccine uptake* (*α*<.05; Table 8). The 2 moderation results overlapped with the mediation results, indicating mixed mediation-moderation relationships [78,80,81].

## Discussion

### Principal Findings

Relatively few studies have sought to predict COVID-19 *vaccine uptake* using machine learning approaches [8,59]. Given that a small set of studies has assessed the psychological basis that may underlie *vaccine uptake* and choices [6,52,53,56,58,59,83], but none have used computational cognition variables based on reward and aversion judgment to predict *vaccine uptake*, we sought to assess whether variables quantifying human judgment predicted *vaccine uptake*. This study found that 7 demographic and 15 judgment variables predicted *vaccine uptake* with balanced and moderate recall and specificity, moderate accuracy, high AUROC, and high precision using a BRF framework. Other machine learning approaches (random forest and logistic regression) produced higher accuracies but lower specificities, indicating a lower prediction of those who did not receive the vaccine. The BRF also had challenges predicting the negative class, as demonstrated by the relatively low NPV despite having higher specificity than random forest and logistic regression. Feature importance analyses from both BRF and random forest showed that the judgment variables collectively dominated the Gini importance scores. Furthermore, demographic variables acted as statistical mediators in the relationship between judgment variables and *vaccine uptake*. These mediation findings support the interpretation of the machine learning results that demographic factors, together with judgment variables, predict COVID-19 *vaccine uptake*.

### Interpretation of Judgment Differences Between Vaccinated and Nonvaccinated Individuals

Those who were fully vaccinated had lower values for loss aversion, ante, peak positive risk, peak negative risk, total reward risk, and total aversion risk, along with higher values for risk aversion, loss resilience, insurance, and trade-off range (refer to Table 1 for variable descriptions). Lower loss aversion corresponds to less overweighting of bad outcomes relative to good ones [84] and a potential willingness to obtain a vaccine with uncertain outcomes. A lower ante suggests that individuals are less willing to engage in risky behaviors surrounding potential infection, which is also consistent with the 4 other judgment variables that define relationships between risk and value (peak positive risk, peak negative risk, total reward risk, and total aversion risk). In participants who indicated full vaccination, lower peak positive risk and peak negative risk were related to individuals having a lower risk that they must overcome to make a choice to either approach or avoid, as per the decision utility equation by Markowitz [39,71]. The lower total reward risk and total aversion risk indicate that the interactions between reward, aversion, and the risks associated with them did not scale significantly; namely, higher reward was not associated with higher risk, and higher negative outcomes were *not* associated with the uncertainty of them. For these participants, the ability of the vaccine to increase the probability of health and reduce the probability of harm from illness did not have to overcome high obstacles in their vaccine choice. Higher risk aversion in vaccinated participants suggests that these participants viewed contracting COVID-19 as a larger risk and, therefore, were more likely to receive the full dose. These findings are consistent with those of a study by Lepinteur et al [58], who found that risk-averse individuals were more likely to accept the COVID-19 vaccination, indicating that the perceived risk of contracting COVID-19 was greater than any risk from the vaccine. Hudson and Montelpare [54] also found that risk aversion may promote vaccine adherence when people perceive contracting a disease as more dangerous or likely. Higher loss resilience in the vaccinated group was also consistent with the perspective that vaccination would improve their resilience and act as a form of insurance against negative consequences. The higher trade-off range suggests that vaccinated individuals have a broader portfolio of preferences and are more adaptive to bad things occurring, whereas a lower trade-off indicates a restriction in preferences and less adaptability in those who did not receive the vaccine.

### Comparison of Prediction Algorithms

When testing these judgment variables (with demographic and COVID-19 precaution behavior variables) in a BRF framework to predict *vaccine uptake*, we observed a high AUROC of 0.79, where an AUROC of 0.8 is often the threshold for excellent model performance in machine learning [85,86]. The similarity of our reported recall and specificity values with the BRF suggests a balance between predicting true positives and true negatives. The high precision indicates a high certainty in predicting those who were fully vaccinated. The BRF model was successful in identifying those who received the full vaccine (positive cases; indicated by high precision and moderate recall) and those who did not (negative cases; indicated by the specificity). However, NPV was low, indicating a higher rate of false prediction of those who did not receive a full dose counterbalanced by a higher specificity that reflects a higher rate of predicting true negatives. These observations are reflected in the moderate accuracy, which measures the number of correct predictions. A comparison of random forest, logistic regression, and BRF revealed that random forest and logistic regression models produced less balance between recall (high) and specificity (low), which could be interpreted as a bias toward predicting the majority class (ie, those who received the vaccine). That being said, the NPV for BRF was lower than that for random forest and logistic regression, where a low NPV indicates a low probability that those predicted to have not received the vaccine truly did not receive the vaccine when taking both classes into account. Together, the results from all 3 machine learning approaches reveal challenges in predicting the negative class (ie, those who did not receive the vaccine). Overall, the 3 models achieved high accuracy, recall, precision, and AUROC. BRF produced a greater balance between recall and specificity, and the outcome of the worst-performing metric (ie, NPV) was still higher than the specificities for the random forest and logistic regression models.

### Feature Importance

Of the 3 prediction algorithms, random forest and BRF had very similar Gini importance results, whereas logistic regression elevated most demographic variables and a minority of judgment variables. This observation could be due to the large variance in each of the judgment variables, which could present challenges for achieving a good fit with logistic regression. In contrast, the demographic and COVID-19 precaution variables had low variance and could be more easily fit in a linear model, hence their significance in the logistic regression results. In comparison to logistic regression, decision trees (eg, BRF and random forest) *use* variable variance as additional information to optimize classification, potentially leading to a higher importance of judgment variables over most demographic and all COVID-19 precaution variables.

Focusing on the model with balanced recall and specificity (ie, the BRF classifiers [with and without COVID-19 precaution behaviors]), the top predictors were 3 demographic variables (age, income, and educational level), with distributions that varied by *vaccine uptake* in manners consistent with those of other reports. Namely, older individuals, those identifying as male and White individuals, and those who indicated a higher income and educational level corresponded to those who were or intended to be vaccinated [2,5,87]. Despite their saliency, these 3 variables together only contributed 23% to the prediction, corresponding to approximately one-third of the contribution from the 15 judgment variables (63%-69%). The individual Gini importance scores for the 15 judgment variables only ranged from 0.039 to 0.049 but were the dominant set of features behind the moderate accuracy, high precision, and high AUROC. The 18% difference between the accuracy and precision measures suggests that variables other than those used in this study may improve prediction, including contextual variables that may influence vaccine choices. Variables may include political affiliation [7], longitude and latitude [8], access to the internet [8], health literacy [54], and presence of underlying conditions [9]. Future work should seek to include these types of variables.

In the second BRF classifier, the 4 COVID-19 precaution behaviors only contributed 6.6% to the prediction. This low contribution could be due to these variables being binary, unlike the other demographic variables, which included a range of categories. In addition, COVID-19 precaution behaviors are specific to the context of the COVID-19 pandemic and do not promote interpretation beyond their specific context. The 15 judgment variables represent a contrast to this as they are empirically computed from a set of functions across many picture categories. An individual with higher risk aversion will generally tolerate higher amounts of uncertainty regarding a potential upside or gain as opposed to settling for what they have. This does not depend on what stimulus category they observe or the stimulus-response condition. Instead, it is a general feature of the bounds to their judgment and is part of what behavioral economists such as Kahneman consider as bounds to human rationality [84].

### Mechanistic Relationships Between Judgment and Demographic Variables

The Gini score plots were clear sigmoid-like graphs (Figure 3), with only 3 of the 7 demographic variables ranking above the judgment variables. This observation was consistent in both BRF classifiers (with and without COVID-19 precaution behaviors), raising the possibility of a statistically mechanistic relationship among the top 3 demographic variables, the 15 judgment variables, and *vaccine uptake*. Indeed, we observed 28 primary mediation effects and 13 secondary mediation effects in contrast to 2 moderation relationships, which also happened to overlap with mediation findings, suggesting mixed mediation-moderation relationships [81,88]. The observation that most judgment variables were significant in mediation relationships but *not* in moderation relationships argues that prediction depended on the directional relationship between judgment and demographic variables to predict *vaccine uptake*. Furthermore, there were more significant primary mediations (when judgment variables were the IVs) compared to secondary mediations, suggesting the importance of judgment variables as IVs and demographic variables as mediators. Mathematically, judgment variables (IVs) influenced *vaccine uptake* (DV), and this relationship was stronger when demographic variables were added to the equation. The 13 secondary mediations all overlapped with the 28 primary mediations, where demographic variables were IVs and judgment variables were mediators, suggesting that demographic variables influenced *vaccine uptake* (DV) and that this relationship became stronger with the addition of judgment variables. This overlap of primary and secondary mediations for 4 of the judgment variables suggests that both judgment and demographic variables influenced the choice of being vaccinated within a mixed mediation framework because adding either one of them to the mediation model regressions made the relationships stronger [49]. The lack of moderation results and a considerable number of overlapping primary and secondary mediation results imply that the relationship between judgment variables and *vaccine uptake* did not depend purely on their interaction with age, income, or educational level (ie, moderation) but, instead, depended on the direct effects of these 3 demographic variables to strengthen the relationship between judgment variables and *vaccine uptake*. This type of analysis of statistical mechanisms is helpful for understanding contextual effects on our biases and might be important for considering how best to target or message those with higher loss aversion, ante, peak positive risk, peak negative risk, total reward risk, and total aversion risk (ie, in those who were not fully vaccinated).

### Model Utility

The developed model is automatable and may have applications in public health. The picture-rating task can be deployed on any smart device or computer, making it accessible to much of the US population or regional populations. The ratings from this task can be automatically processed, and the results can be stored in local or national databases. This method of data collection is novel in that persons cannot bias their responses as the rating task has no perceivable relation to vaccination choices. Government and public health bodies can access these data to determine predicted *vaccine uptake* rates locally or nationally, which can be used to (1) prepare vaccine rollouts and supply chain demand, (2) prepare health care institutions in areas that may experience low vaccine adherence and potentially higher infection rates, and (3) determine which areas may need more targeted messaging to appeal to specific judgment profiles. For use case 3, messaging about infection risks or precaution behaviors could be framed to address those with lower risk aversion, who, in this study, tended to forgo vaccination. Given that such individualized data would not be available a priori, it would be more plausible to collect data from similarly sized cohorts in geographic regions of concern to obtain regional judgment behavior profiles and, thus, target use cases 1 to 3. Further development of this model with different population samples might also improve our understanding of how certain judgment variables may be targeted with different types of messaging, offering a means to potentially improve *vaccine uptake*. This model might also be applied to other mandated or recommended vaccines such as those for influenza or human papillomavirus, ultimately improving preparation and messaging efforts. However, future work would be needed to model these varying vaccine choices.

Given the use of demographic variables in the proposed model, specific demographic populations could be assessed or considered for messaging. If particular demographic groups are predicted to have a low *vaccine uptake* rate, messaging can be targeted to those specific groups. For example, we observed that a higher percentage of female individuals were not fully vaccinated when compared to male individuals. This could be related to concerns about the COVID-19 vaccine affecting fertility or pregnancy. To improve uptake in this population, scientifically backed messaging could be used to confirm the safety of the vaccine in this context. Lower rates of vaccination have been reported in Black communities, which was also observed in this study. Researchers have identified targetable issues related to this observation, which include engagement of Black faith leaders and accessibility of vaccination clinics in Black communities, to name a few [89].

In summary, this model could be used to predict *vaccine uptake* at the local and national levels and further assess the demographic and judgment features that may underlie these choices.

### Limitations

This study has a number of limitations that should be considered. First, there are the inherent limitations of using an internet survey—namely, the uncontrolled environment in which participants provide responses. Gold Research, Inc, and the research team applied stringent exclusion criteria, including the evaluation of the judgment graphs given that random responses produce graphs with extremely low *R*^2^ fits (eg, <0.1). This was not the case in our cohort of 3476 participants, but this cannot perfectly exclude random or erroneous responses to other questionnaire components. Second, participants with mental health conditions were oversampled to meet the criteria for other survey components not discussed in this paper. This oversampling could potentially bias the results, and future work should use a general population sample to verify these findings. Third, demographic variability and the resulting confounds are inherent in population surveys, and other demographic factors not collected in this study may be important for prediction (eg, religion and family size). Future work might consider collecting a broader array of demographic factors to investigate and include in predictive modeling. Fourth, we used a limited set of 7 demographic variables and 15 judgment variables; however, a larger set of judgment variables is potentially computable and could be considered for future studies. There is also little information on how post–COVID-19 effects, including socioeconomic effects, affect COVID-19 vaccination choices.

### Conclusions

To our knowledge, there has been minimal research on how biases in human judgment might contribute to the psychology underlying individual vaccination preferences and what differentiates individuals who were fully vaccinated against COVID-19 from those who were not. This population study of several thousand participants demonstrated that a small set of demographic variables and 15 judgment variables predicted *vaccine uptake* with moderate to high accuracy and high precision and AUROC, although a large range of specificities was achieved depending on the classification method used. In an age of big data machine learning approaches, this study provides an option for using fewer but more interpretable variables. Age, income, and educational level were independently the most important predictors of *vaccine uptake*, but judgment variables collectively dominated the importance rankings and contributed almost two-thirds to the prediction of COVID-19 vaccination for the BRF and random forest models. Age, income, and educational level significantly mediated the statistical relationship between judgment variables and *vaccine uptake*, indicating a statistically mechanistic relationship grounding the prediction results. These findings support the hypothesis that small sets of judgment variables might provide a target for vaccine education and messaging to improve uptake. Such education and messaging might also need to consider contextual variables (ie, age, income, and educational level) that mediate the effect of judgment variables on *vaccine uptake*. Judgment and demographic variables can be readily collected using any digital device, including smartphones, which are accessible worldwide. Further development and use of this model could (1) improve *vaccine uptake*, (2) better prepare vaccine rollouts and health care institutions, (3) improve messaging efforts, and (4) have applications for other mandated or government-recommended vaccines.

## Appendix

Multimedia Appendix 1 Supplementary material.

Multimedia Appendix 2 Data set.

## Abbreviations

AUROC: area under the receiver operating characteristic curve
BRF: balanced random forest
DV: dependent variable
IV: independent variable
NPV: negative predictive value
RPT: relative preference theory

## Data Availability

The data set and corresponding key used in this study are available in Multimedia Appendix 2.
