# Supplementary material for: Predicting COVID-19 Vaccination Uptake Using a Small and Interpretable Set of Judgment and Demographic Variables: Cross-Sectional Cognitive Science Study
Source: JMIR Public Health Surveill. 2024 Mar 18;10:e47979. doi: 10.2196/47979 (PMC10953811; doi:10.2196/47979)
Supplement: Multimedia Appendix 1 [file publichealth_v10i1e47979_app1.docx]

**Supplemental Material**

**Methods: RPT Analysis and Quality Assurance**

Quantified variables included the average magnitude (*K*), variance (*σ*), and the pattern or information (i.e., Shannon entropy (*H*)) related to participants’ preference behavior [35]. *K* reflected the average (mean) of positive ratings a subject made ($K_{+}$) or negative ratings ($K_{-}$) within each picture category. Other metrics included the variance in positive ratings ($\sigma_{+}$) or negative ratings ($\sigma_{-}$), along with the Shannon entropy (i.e., information; see Shannon & Weaver, 1949 [35]) of positive ratings ($H_{+}$) or negative ratings ($H_{-}$) for stimuli within each category. The Shannon entropy is a core variable in information theory that characterizes the degree of uncertainty across a set of responses [35]; it quantifies the pattern of judgements made to a set of stimuli and could thus be considered a memory variable. Collectively, these variables capture judgments about the valence of judgement (positive vs. negative or approach vs. avoidance) as well as its magnitude (intensity of rating) to describe relative preferences (**Fig. 1**) [18,64,36,33]

When evaluating data quality, raw data was assessed for cases when *K*=0 for a given category (i.e., cases where the subject made all neutral ratings to neither approach nor to avoid any stimulus in the category). Computing the Shannon entropy, *H*, for a given picture category requires that *K*>0 given that when *K*=0, the *H* computation results in evaluating log_10_(0/0), which is undefined. In these cases, the Shannon entropy was set to *H*=0 for categories in which the subject rated “0” for all the stimuli.

Before carrying out the RPT analyses, and fitting models to participants’ ratings, data was further screened for additional criteria beyond when *K*=0 for a given category. The complete set of model fit inclusion/exclusion criteria was as follows:

1. Valid entropy (*H*) calculations (see prior paragraph),
2. Exclusion of extreme outliers (loss aversion values > 200, resulting in N=42 exclusions and positive quadratic area > 100, resulting in N=5 exclusions).
3. Sufficient number of data points to fit the model with a computable R^2^ (e.g., at least three points for a non-linear fit), and
4. Coherence of model fits between individual and group data. This last criterion required that the curve direction for individual subject fits be consistent with the curve direction of the group-level statistical fits (and boundary envelopes), and therefore corroborate most of the observed subject data.

Criteria (3) and (4) are necessary operational definitions for quality assurance given the potential for convergence failures with curve fitting.

In total, six types of model fitting were performed for the rating data: group and individual models for the (*K, H*) data, (*K, σ*) data, and ($H_{+},H_{-}$) data distributions. For the group data, we generated group-level data fits along with boundary envelopes (power-law fits and logarithmic fits for group (*K, H*) data), and quadratic fits for group (*K, σ*) data to guide the focus of statistical testing based on the power law fits (*K, H*), and quadratic fits (*K, σ*) for individual data. Individual data then followed these fits based on logarithmic and simple power-law fits for individual (*K, H*) value functions, quadratic fits for individual (*K, σ*) limit functions, and radial fits for individual ($H_{+},H_{-}$) tradeoff distributions [18,36].

**Methods: Complete judgment variable (RPT) descriptions**

These descriptions have been outlined in Azcona et al., 2022 [11]. This analysis used a feature space characterizing approach and avoidance judgments [18,64,36,33]. Each participant’s rating behavior was modeled to produce approach/avoidance variables of mean magnitude (*K*), variance or standard deviation ($\sigma$), and uncertainty from the pattern of ratings (Shannon entropy, *H*). These variables were then plotted as *KH*, *K*$\sigma$, and $H_{+}H_{-}$ graphs. $K_{+}H_{+}$ and $K_{+}\sigma_{+}$ are plotted separately from $K_{-}H_{-}$ and $K_{-}\sigma_{-}$*,* thereby calibrating approach/avoidance, *K*, to the pattern of prior judgments, *H*, and their variance, $\sigma$. These three variables (*K, H,* $\sigma$) show consistent functions in their relationship, two of which resemble graphs observed in prospect theory [38,65,84] and another two of which resemble the graphs for the risk-reward function in portfolio theory [18,36,33,66]. These values were plotted as curves (see **Fig. 1B, Fig. S2**) in MATLAB using the library *polyfit* for each [18,64,36,33].

An array of graph features was extracted from each curve that can be interpreted psychologically. The features used from these graphs are described below in psychological terms and organized relative to the curve from which they were extracted.

(1) The (*K, H*) curve consists of ($K_{+}$*,* $H_{+}$) on the positive x-axis and ($K_{-}$*,* $H_{-}$) on the negative x-axis; it is analogous to the value function from Kahneman’s Prospect Theory [38,65,84]. The RPT features that can be extracted from this curve include *Risk Aversion, Loss Resilience, Loss Aversion, Ante*, and *Insurance.*

*Loss Aversion*: *Loss Aversion* (LA) is the absolute value of the ratio of the linear regression slope of $\left( \log K_{-},\log H_{-} \right)$ to the linear regression slope of $\left( \log K_{+},\log H_{+} \right)$. It intuitively measures the degree to which an individual person overweighs losses to gains. LA a fundamental measure in Kahneman and Tversky’s (1979) [38,84] prospect theory, which informally states that humans have a cognitive bias to overweight losses relative to gains in the presence of uncertainty.

*Risk Aversion*: *Risk Aversion* (RA) is extracted as the ratio of the second derivative of the $\left( H_{+}\boldsymbol{,}K_{+} \right)$ curve to its first derivative, which also produces a curve. To produce a unitary value for comparison across cohorts, we calculated RA for $K_{+}=0.5$. Informally, RA measures the degree to which an individual prefers a likely reward in comparison to a better more uncertain reward. RA is a common notion in economics that studies decision-making under uncertainty [67].

*Loss Resilience*: *Loss Resilience* (LR) is defined as the absolute value of the ratio of the second derivative of the ($K_{-}$*,* $H_{-}$) curve to its first derivative, which also produces a curve. For prediction, we calculated *Loss Aversion* at $K_{-}$ = 1.5. Informally, LR is the degree to which an individual prefers to lose a small defined amount in comparison to losing a greater amount with more uncertainty associated with this loss.

*Ante*: *Ante* is the value of $K_{+}$ when setting $H_{+}=0$. This intuitively measures the ante one needs to engage in a game of chance and models the amount of a bid an individual is willing to make to enter a game of chance (e.g., poker).

*Insurance*: *Insurance* is the value of $K_{-}$ when setting $H_{-}$ = 0. It intuitively measures how much insurance an individual might need against bad outcomes. It mirrors the ante, but in the framework of potential losses.

(2) The (*K,* $\sigma$) curve consists of ($K_{+}$*,* $\sigma_{+}$) on the positive x-axis and ($K_{-}$*,* $\sigma_{-}$) on the negative x-axis; it is analogous to the mean-variance curve derived from portfolio theory [71]. This allows comparison of preference magnitude (i.e., *K*) with respect to variance in rewards and sanctions. The (*K,* $\sigma$) curve models the following question: Would an individual prefer a dollar with probability one, or value drawn from a normal distribution with mean of two and variance of two? The RPT features that are extracted from this curve include *Peak Positive Risk, Peak Negative Risk, Reward Tipping Points, Aversion Tipping Point, Total Reward Risk, and Total Aversion Risk.*

*Peak Positive Risk*: *Peak Positive Risk* (Peak PR) is the value of $\sigma_{+}$ for the derivative $\frac{d\sigma_{+}}{dK_{+}}=0$. Intuitively, this represents the maximum variance for approach behavior. In this sense, $\sigma_{+}$ models where increases in positive value transition from a relationship with increases in risk, to a relationship with decreases in risk. Markowitz described decision utility similarly, so that the positive apex models when variance changes from weighing against a decision to facilitating a decision [71].

*Peak Negative Risk*: The *Peak Negative Risk* (Peak NR) is value of $\sigma_{-}$ where the derivative $\frac{{d\sigma}_{-}}{{dK}_{-}}$ = 0. Intuitively, this represents the maximum variance for avoidance behavior. Like with the *Peak Positive Risk*, this transition point is important to consider for avoidance decisions in the context of Markowitz’s decision utility [71].

*Reward Tipping Point*: The *Reward Tipping Point* (Reward TP) is the value of $K_{+}$when the derivative $\frac{d\sigma_{+}}{dK_{+}}=0$. Intuitively, this represents the rating intensity with maximum variance for approach behavior, potentially when an individual decides to approach a goal-object.

*Aversion Tipping Point*: The *Aversion Tipping Point* (Aversion TP) is the value of$K_{-}$ where the derivative $\frac{{d\sigma}_{-}}{{dK}_{-}}$ = 0. Intuitively, this represents the rating intensity with maximum variance for approach behavior, potentially when an individual decides to avoid a goal-object.

*Total Reward Risk*: The *Total Reward Risk* (Total RR) is the area under the curve (AUC) of the first quadrant of the $\left( K_{+}\boldsymbol{,}\sigma_{+} \right)$. This variable represents the relationship between $K_{+}$ and $\sigma_{+}$and can be thought of as a quantity that measures the amount of value an individual associates to positive stimuli.

*Total Aversion Risk*: The *Total Aversion Risk* (Total AR) is the area under the curve on the negative quadrant of the graph of ($K_{-}$*,*$\sigma_{-}$). Intuitively, this variable represents the relationship between$K_{-}$ and $\sigma_{-}$and can be thought of as a quantity that measures the amount of overall value a person associates to a negative stimulus.

(3) The ($H_{+}$***,*** $H_{-}$) curve allows the comparison of patterns in approach and avoidance judgments. The RPT features that are extracted from this curve include *Reward Aversion Tradeoff*, *Tradeoff Range, Reward Aversion Consistency,* and *Consistency Range.*

*Reward Aversion Tradeoff*: The *Reward Aversion Tradeoff* (RA Tradeoff) is the mean of the polar angles of the points in the ($H_{+}$*,* $H_{-}$) plane. Intuitively, this measures the balance between patterns in approach and avoidance behavior.

*Tradeoff Range*: This is the standard deviation of the polar angles of the points in the ($H_{+}$*,* $H_{-}$) plane. Intuitively, this measures the standard deviation in the patterns of approach and avoidance behavior. This variance represents the spread for positive preferences and negative preferences across a set of potential goal-objects and is one measure of the breadth of an individual’s (or group’s) preferences. Intuitively, this describes the breadth of an individual’s portfolio of preferences.

*Reward Aversion Consistency*: The *Reward Aversion Consistency* (RA Consistency) measures the mean of the distances of the data points in the ($H_{+}$*,* $H_{-}$) curve to the origin. Intuitively, this defines how individuals can have strong preferences (i.e., biases) for the same thing, reflecting *conflict*, or having low preferences for something, reflecting *indifference*. This gets at the consistency or compatibility of approach and avoidance, and how you can both like and dislike something, or be indifferent to both its positive and negative features.

*Consistency Range*: The *Consistency Range* measures the standard deviation of the distances of the data points in the ($H_{+}$*,* $H_{-}$) plane to the origin. Intuitively, this measures how the points in the *HH* plane vary with regard to the radial distance from the origin. The variance in this radial distance will reflect how much an individual goes between having *conflicting* preferences versus having *indifferent* ones.

**Methods: Mediation and Moderation**

The procedure for mediation was implemented following Iacobucci et al. 2012 [68] with logistic regression implemented for steps 1 and 3, and linear regression for step 2 (Y=dependent variable, X=independent variable, M=mediator variable). The mediators (age, income and education level) were treated as continuous variables and vaccination status was treated as binary variable.

$$Step 1 :Y= \gamma_{1}+ c\left( X \right)+\epsilon_{1}$$

$$Step 2 :M=\gamma_{2}+ a\left( X \right)+\epsilon_{2}$$

$$Step 3 : Y= \gamma_{3}+ c^{'}\left( X \right)+ b(M)+\epsilon_{3}$$

Using the parameter estimates and the standard errors of the coefficients from above equation, *z_mediation_* and *p_overall_* were calculated.

$$z_{a}=\frac{a}{s_{a}}$$

$$z_{b}=\frac{b}{s_{b}}$$

$$z_{mediation}=\frac{z_{ab}}{\sigma_{z_{ab}}}=\frac{z_{a}z_{b}}{\sigma_{z_{ab}}}$$

$$z_{mediation}=\frac{\frac{a}{s_{a}}\times\frac{b}{s_{b}}}{\sqrt{z_{a}^{2}+z_{b}^{2}+1}}$$

*p_overall_* was calculated by placing *z_mediation_* in a normal distribution.

The moderation model proposes that the strength and direction of the relationship between X (independent variable) and Y (dependent variable) is controlled by the moderator variable, Mo. The moderation is characterized by the interaction term between X and Mo in binomial logistic regression as given below:

$$log(odds)= \beta_{0}+\beta_{1}X+\beta_{2}Mo+\beta_{3}\left( X*Mo \right)+\epsilon$$

Moderation is significant if $p_{\beta_{3}}\leq.05$ (the interaction term) and $p_{overall}\leq.05$ (for the overall model).

To check if the overall model was significant, we built full and null models and used the Likelihood Ratio test to determine if the null hypothesis was rejectable.

Full model:

$$log(odds)= \beta_{0}+\beta_{1}X+\beta_{2}Mo+\beta_{3}\left( X*Mo \right)+\epsilon$$

Null Model:

$$log(odds)= \beta_{0}+\epsilon$$

Null hypothesis:

$$H_{0}:\beta_{1}=\beta_{2}=\beta_{3}=0$$

Alternative hypothesis:

$$H_{A}:\beta_{i}\neq0\boldsymbol{;}for atleast one \beta_{i};where i=1,2,3$$

To test if the coefficient of interaction term ($\beta_{3})$was significant, we built full and restricted models and used the Likelihood Ratio test to determine if the null hypothesis was rejectable.

Full model:

$$\boldsymbol{l}og(odds)= \beta_{0}+\beta_{1}X+\beta_{2}Mo+\beta_{3}\left( X*Mo \right)+\epsilon$$

Restricted Model:

$$log(odds)= \beta_{0}+\beta_{1}X+\beta_{2}Mo+\epsilon$$

Null hypothesis:

$$H_{0}:\beta_{3}=0$$

Alternative hypothesis:

$$H_{A}:\beta_{3}\neq0$$

**References**

11. Azcona EA, Kim BW, Vike NL, Bari S, Lalvani S, Stefanopoulos L, et al. Discrete, recurrent, and scalable patterns in human judgement underlie affective picture ratings. arXiv. Preprint posted online March 12, 2022. 2022.:2203.06448

18. Kim BW, Kennedy DN, Lehár J, Lee MJ, Blood AJ, Lee S, et al. Recurrent, robust and scalable patterns underlie human approach and avoidance. PLoS One. May 26, 2010;5(5):e10613. [doi: 10.1371/journal.pone.0010613]

33. Viswanathan V, Sheppard JP, Kim BW, Plantz CL, Ying H, Lee MJ, et al. A quantitative relationship between signal detection in attention and approach/avoidance behavior. Front Psychol. Feb 21, 2017;8:122. [doi: 10.3389/fpsyg.2017.00122]

35. Shannon CE, Weaver W. The Mathematical Theory of Communication. Urbana, IL. The University of Illinois Press; 1949. [doi: 10.3389/fnins.2017.00136]

36. Livengood SL, Sheppard JP, Kim BW, Malthouse EC, Bourne JE, Barlow AE, et al. Keypress-based musical preference is both individual and lawful. Front Neurosci. May 02, 2017;11:136. [doi: 10.3389/fnins.2017.00136]

38. Kahneman D, Tversky A. Prospect theory: an analysis of decision under risk. Econometrica. Mar 1979;47(2):263-292. [doi: 10.2307/1914185]

64. Lee S, Lee MJ, Kim BW, Gilman JM, Kuster JK, Blood AJ, et al. The commonality of loss aversion across procedures and stimuli. PLoS One. Sep 22, 2015;10(9):e0135216. [doi: 10.1371/journal.pone.0135216]

65. Tversky A, Kahneman D. Advances in prospect theory: cumulative representation of uncertainty. J Risk Uncertainty. Oct 1992;5(4):297-323. [doi: 10.1007/BF00122574]

66. Sheppard JP, Livengood SL, Kim BW, Lee MJ, Blood AJ. Connecting prospect and portfolio theories through relative preference behavior. In: Proceedings of the 14th Annual Meeting on Society for NeuroEconomics. 2016. Presented at: SNE '16; August 28-30, 2016;102-103; Berlin, Germany. URL: https://staging.neuroeconomics.org/wp-content/uploads/2016/ 08/AbstractBookSNE2016.pdf

67. Zhang R, Brennan TJ, Lo AW. The origin of risk aversion. Proc Natl Acad Sci U S A. Dec 16, 2014;111(50):17777-17782. [doi: 10.1073/pnas.1406755111]

68. Iacobucci D. Mediation analysis and categorical variables: the final frontier. J Consum Psychol. Apr 12, 2012;22(4):582-594. [doi: 10.1016/J.JCPS.2012.03.006]

71. Markowitz H. The Utility of Wealth. J Political Econ. Apr 1952;60(2):151-158. [doi: 10.1086/257177]

84. Kahneman D, Tversky A. On the interpretation of intuitive probability: a reply to Jonathan Cohen. Cognition. Jan 1979;7(4):409-411. [doi: 10.1016/0010-0277(79)90024-6]

**Supplementary Tables and Figures**

**Figure S1**. Questionnaire of diagnosed conditions used for quality assurance.


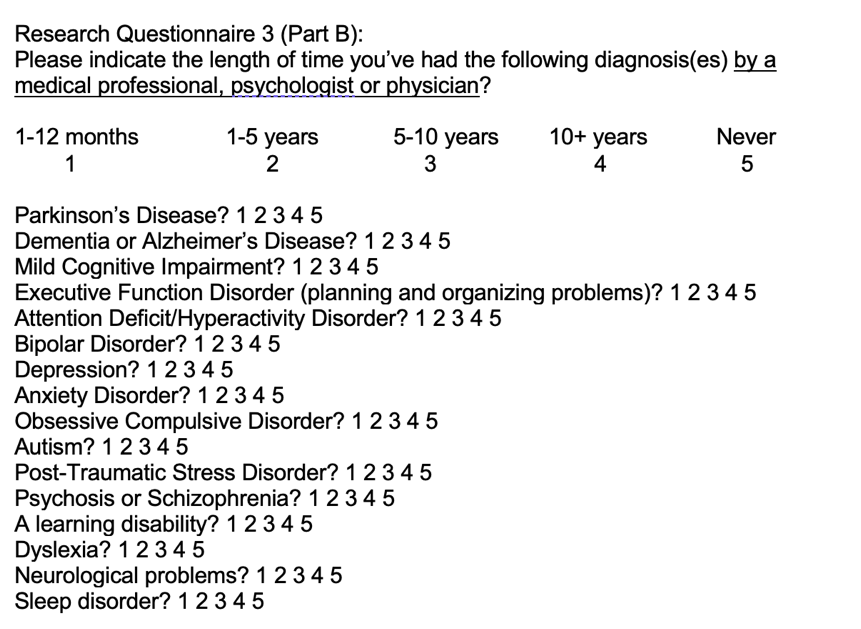


**Table S1.** COVID-19 precaution behavior questions with abbreviated terms.

**Table S2**. Response sizes for COVID-19 precaution behavior questions.

**Figure S2.** Representative fittings for each of the functions. (A) Picture rating data from 500 randomly sampled participants were fit using the value function. (B) Picture rating data from the same 500 randomly sampled participants were fit using the limit function. (C) Picture rating data from the same 500 randomly sampled participants were fit using the tradeoff function. (D) Goodness of fit was evaluated for the value and limit functions. The mean and standard deviation (S.D.) were reported for R^2^, adjusted R^2^, and F-statistic.


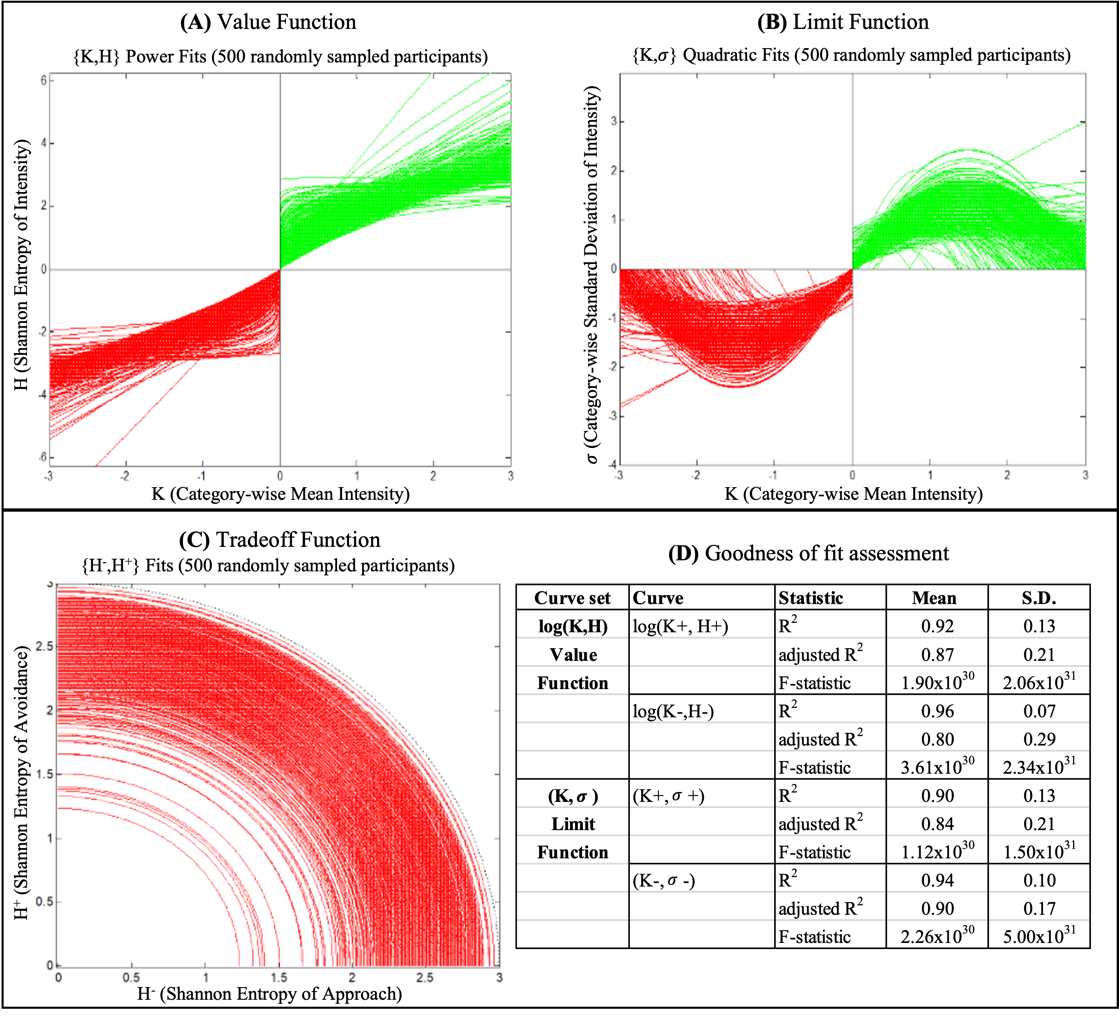


**Table S3**. Judgment variable summary statistics. Median, mean, S.D. (of the mean), and 95% confidence intervals (5% and 95%) were determined for each of the 15 mathematically-derived judgment variables following quality assurance.

**Table S4.** Balanced random forest results using the top three predictors [age, income, and education level (edu)]. AUC ROC = area under the receiving operating characteristics curve.

**Table S5.** Balanced random forest results using the top three predictors [age, income, and education level (edu)] and 15 judgment variables. AUC ROC = area under the receiving operating characteristics curve.
